# Supplementary material for: Warming of hot extremes alleviated by expanding irrigation
Source: Nat Commun. 2020 Jan 15;11:290. doi: 10.1038/s41467-019-14075-4 (PMC6962396; doi:10.1038/s41467-019-14075-4)
Supplement: Supplementary file 1 — Supplementary Information [file 41467_2019_14075_MOESM1_ESM.pdf]

Supplementary Information - Warming of hot extremes alleviated by expanding irrigation

Thiery et al.

Supplementary Figures

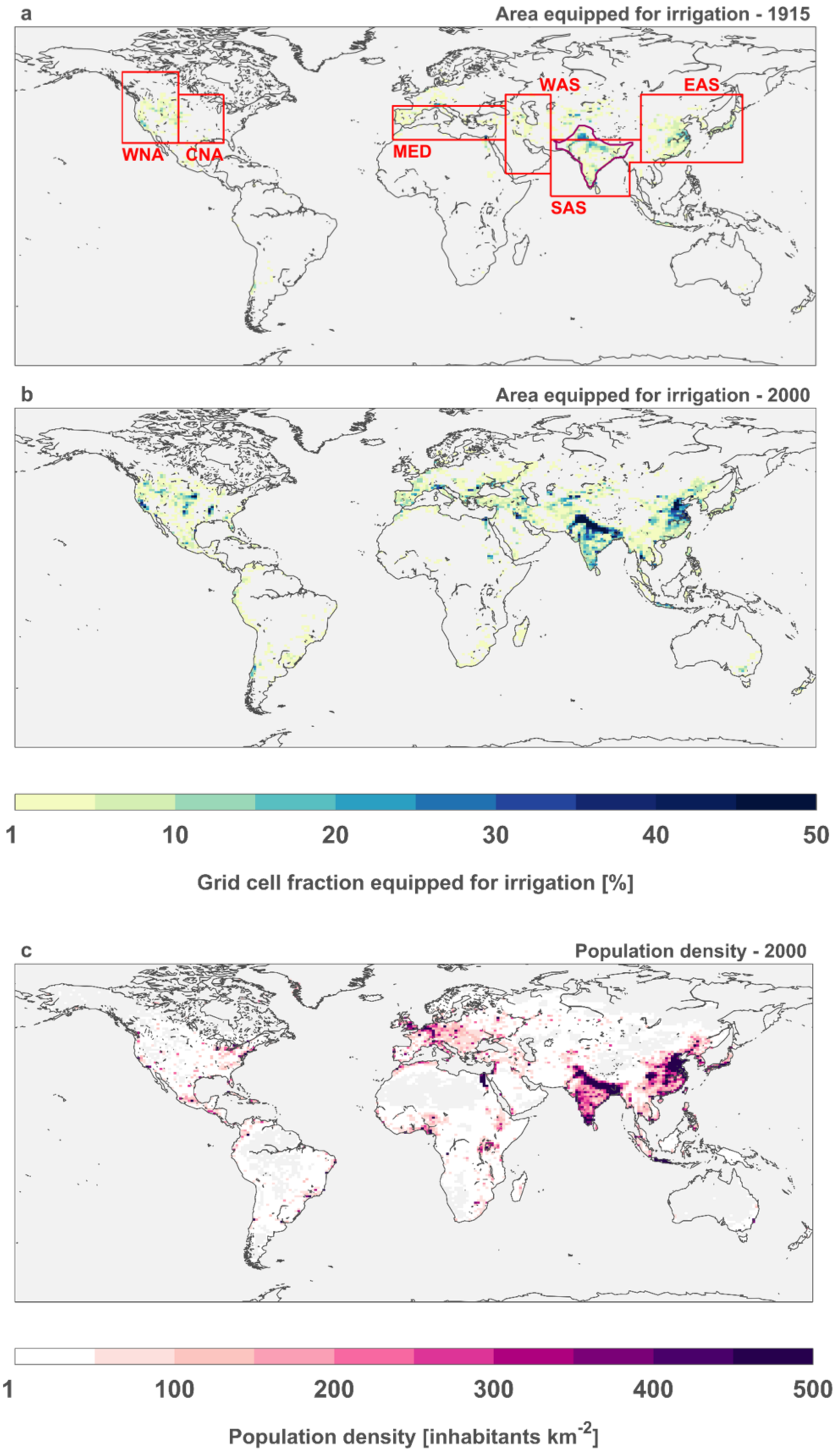

Supplementary Figure 1 | Irrigation expansion during the 20th century. Percentage of each grid cell equipped for irrigation in 1915 (**a**) and 2000 (**b**) from the Historical Irrigation Data set (HID)[1]. (**c**) Population density in 2000 from the Gridded Population of the World (GPW) version 4 data set[2]. The purple polygon in panel (a) marks the countries Pakistan, India, Nepal and Bangladesh, whereas red rectangles mark the regions western North America (WNA), central North America (CNA), southern Europe and Mediterranean (MED), West Asia (WAS), South Asia (SAS), and East Asia (EAS) used in Supplementary Fig. 7 and originally defined in the Special Report on Managing the Risks of Extreme Events (SREX)[3].

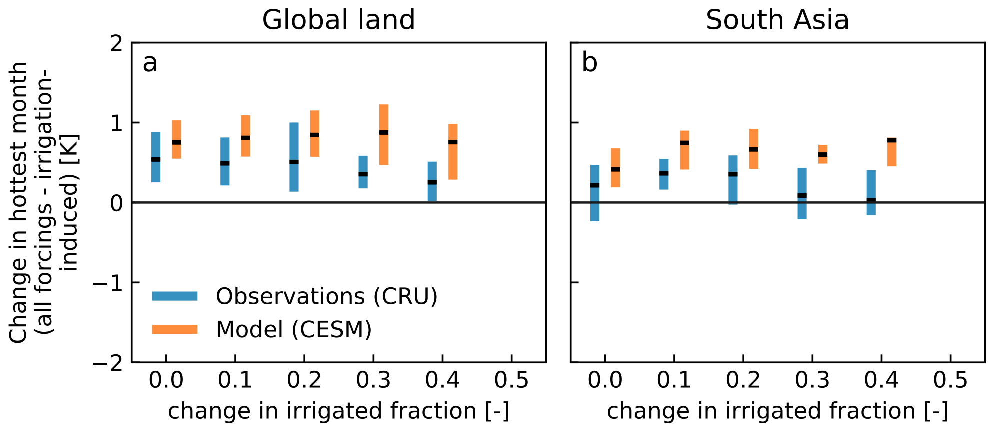

Supplementary Figure 2 | Negative relation largely removed after correcting for irrigation-induced cooling. Same as Fig. 1 (**a-b**), but now showing the difference between the total (ΔTXm) and irrigation-induced ($\Delta TXm_{\mathrm{irr}}$) change in average daily maximum temperature during the hottest month of the year.

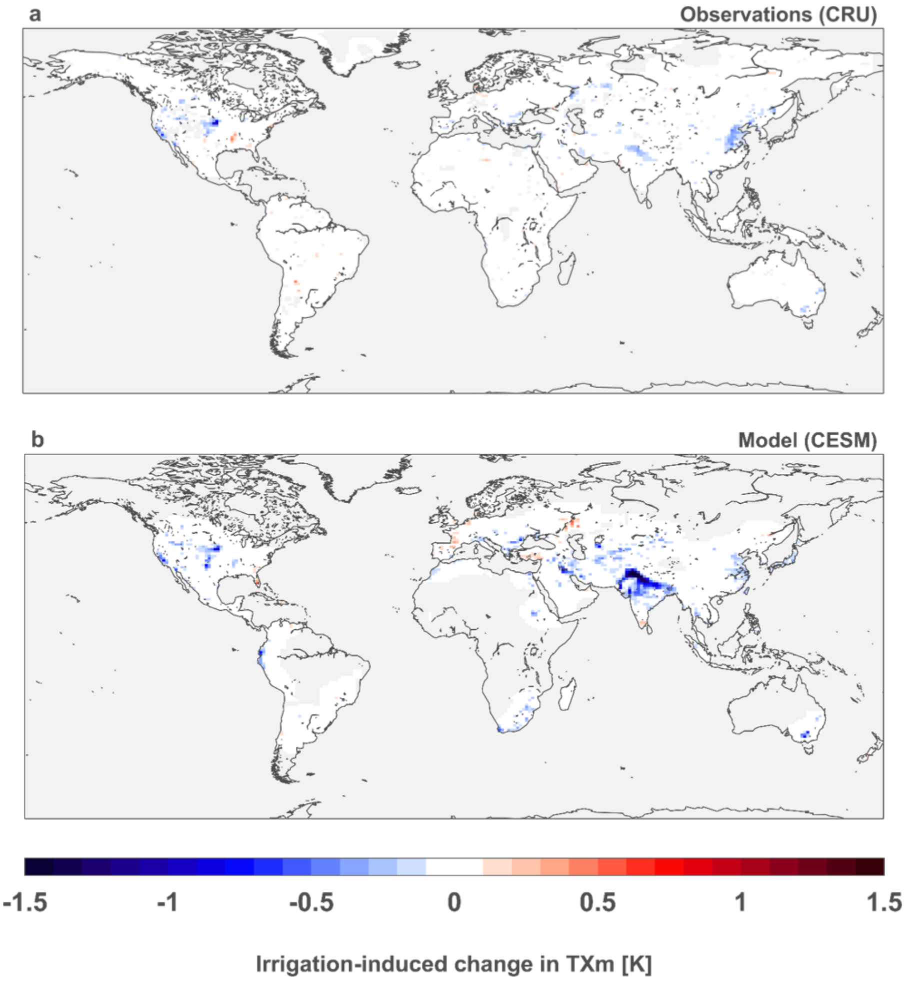

Supplementary Figure 3 | Consistent representation of irrigation-induced cooling in observations and model simulations. Effect of irrigation expansion throughout the 20th century on average daytime temperatures during the hottest month of the year ($\Delta TXm_{\mathrm{irr}}$) from CRU TS v4.02 gridded observations (**a**) and CESM climate simulations (**b**), as computed by the window searching algorithm (see Methods). In case of CESM, the algorithm was applied to the IRR_20C and IRR ensembles.

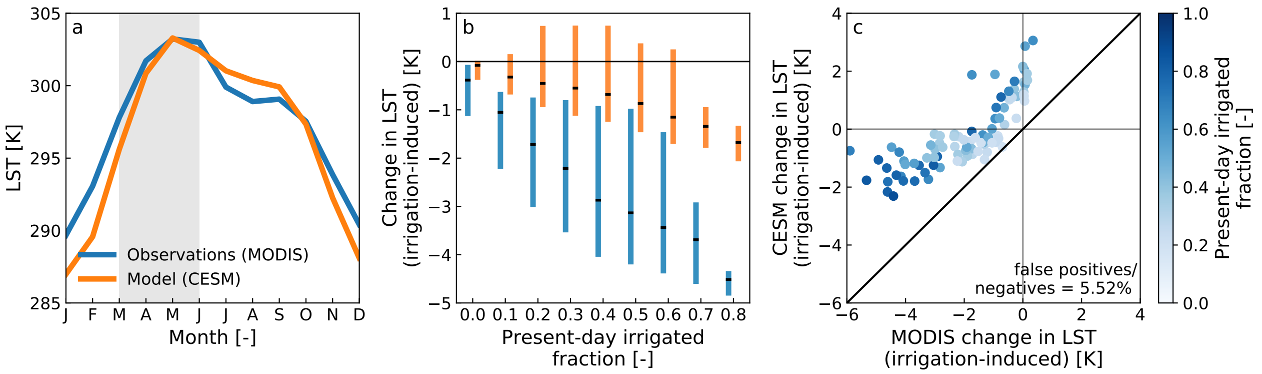

Supplementary Figure 4 | Observed and simulated land surface temperatures affected by irrigation. Seasonal cycle in observed and simulated absolute land surface temperatures (LST) across South Asia (**a**), irrigation-induced change in present-day absolute LST as a function of present-day irrigated fraction (**b**), and scatter plot of present-day simulated versus observed irrigation-induced change in LST (**c**). The window searching algorithm was applied during the months March-May (MAM; grey band in panel **a**). Blue lines and boxplots represent results from the MODIS observational data, orange lines and boxplots show results from the CESM global climate simulations under present-day climate conditions (grid-scale analysis). Boxplots indicate the spatial distribution (center line: median; box limits: upper and lower quartiles; whiskers and outliers: not shown).

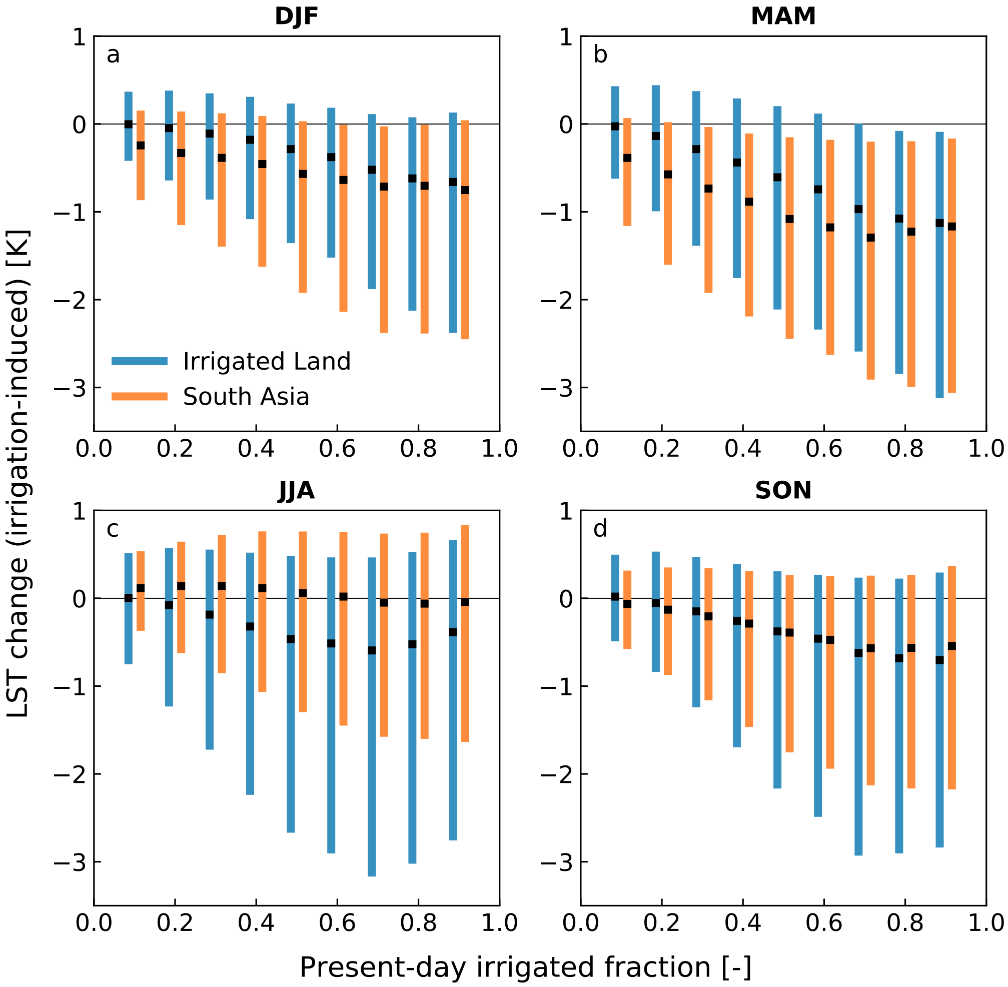

Supplementary Figure 5 | Seasonal signature of irrigation influence on observed land surface temperatures. Irrigation-induced change in present-day absolute land surface temperature (LST) as a function of present-day irrigated fraction for December-January (DJF) (**a**), March-May (MAM) (**b**), June-August (JJA) (**c**), and September-November (SON) (**d**). Blue bars represent results for irrigated land, whereas orange bars show results for South Asia. Irrigated land is defined here as all pixels with >10% irrigated crop fraction, and corresponds to ∼5% of all land area. South Asia is defined as Pakistan, India, Nepal and Bangladesh, and represents ∼3% of all land. Boxplots indicate the spatial distribution (center line: median; box limits: upper and lower quartiles; whiskers and outliers: not shown).

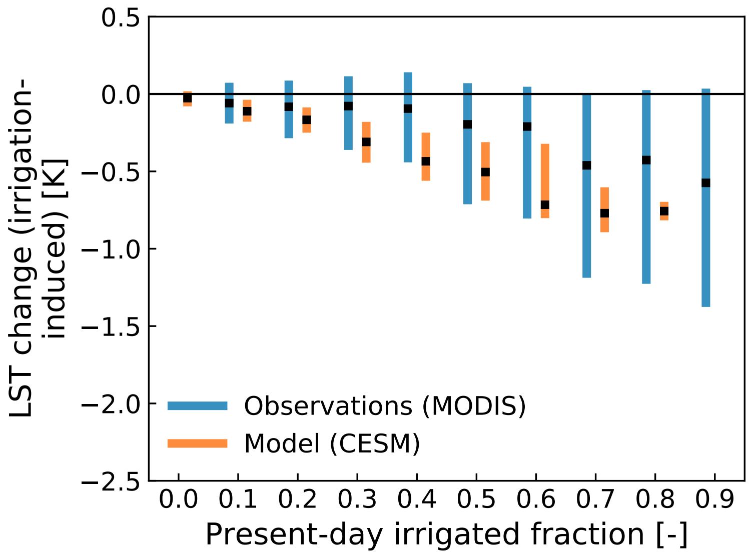

Supplementary Figure 6 | Comparison of irrigation effects on land surface temperatures as derived from subgrid-scale CESM output and MODIS data. Irrigation-induced change in land surface temperature (LST) across South Asia as derived from applying the window-searching method to MODIS (blue) and from scaling the subgrid-scale LST difference between irrigated and rainfed crop tiles by the present-day irrigated fraction (orange). Results are representative of annual conditions during 2003-2010. Boxplots indicate the spatial distribution (center line: median; box limits: upper and lower quartiles; whiskers and outliers: not shown).

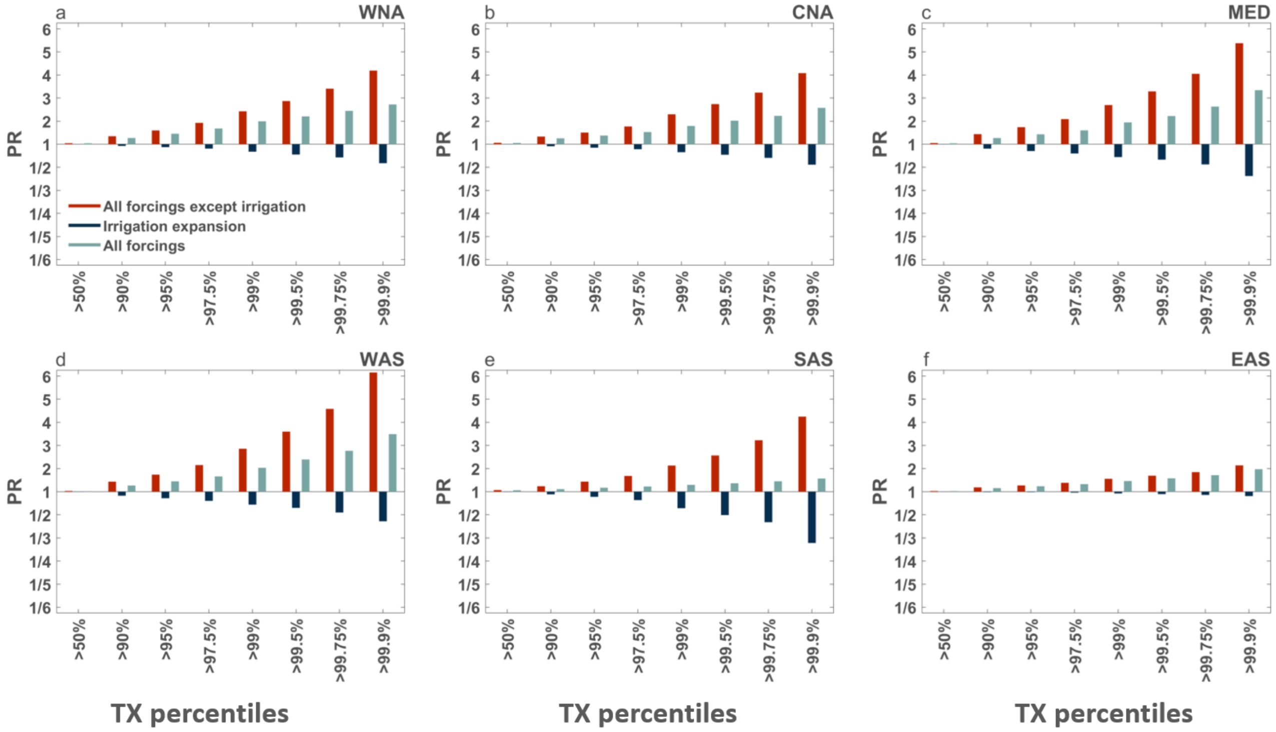

Supplementary Figure 7 | Regional masking of trends in hot extremes due to irrigation. Same as Fig. 3, except for SREX regions western North America (WNA, **a**), central North America (CNA, **b**), southern Europe and Mediterranean (MED, **c**), West Asia (WAS, **d**), South Asia (SAS, **e**) and East Asia (EAS, **f**)[3].

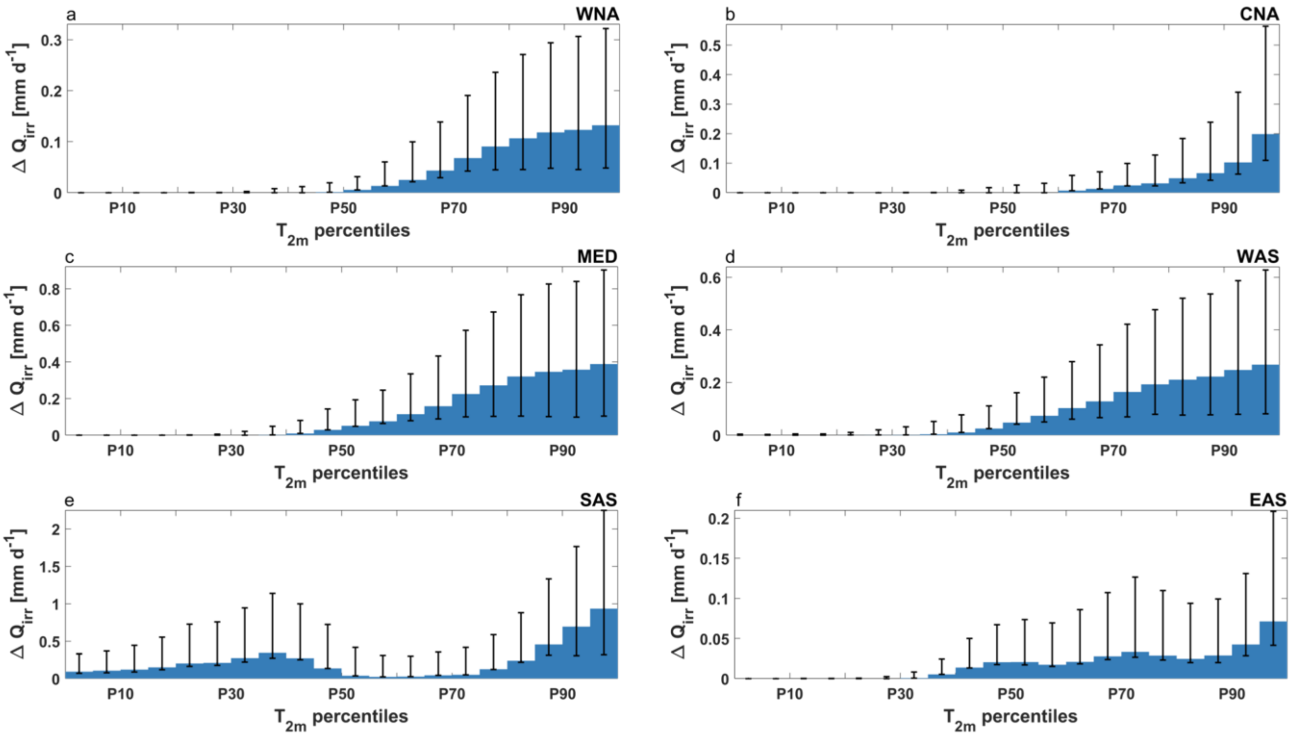

Supplementary Figure 8 | Simulated present-day irrigation rates Q_{irr} as a function of temperature in SREX regions western North America (WNA, **a**), central North America (CNA, **b**), southern Europe and Mediterranean (MED, **c**), West Asia (WAS, **d**), South Asia (SAS, **e**) and East Asia (EAS, **f**)[3]. Q_{irr} was binned according to daily mean temperature using a bin width of 5%; bar heights indicate the change in bin median and whiskers the change in 25th and 75th percentile of spatially averaged, daily values. The binning was performed on the individual IRR ensemble members and bin statistics were subsequently averaged over all ensemble members. Note the varying y-axis range.

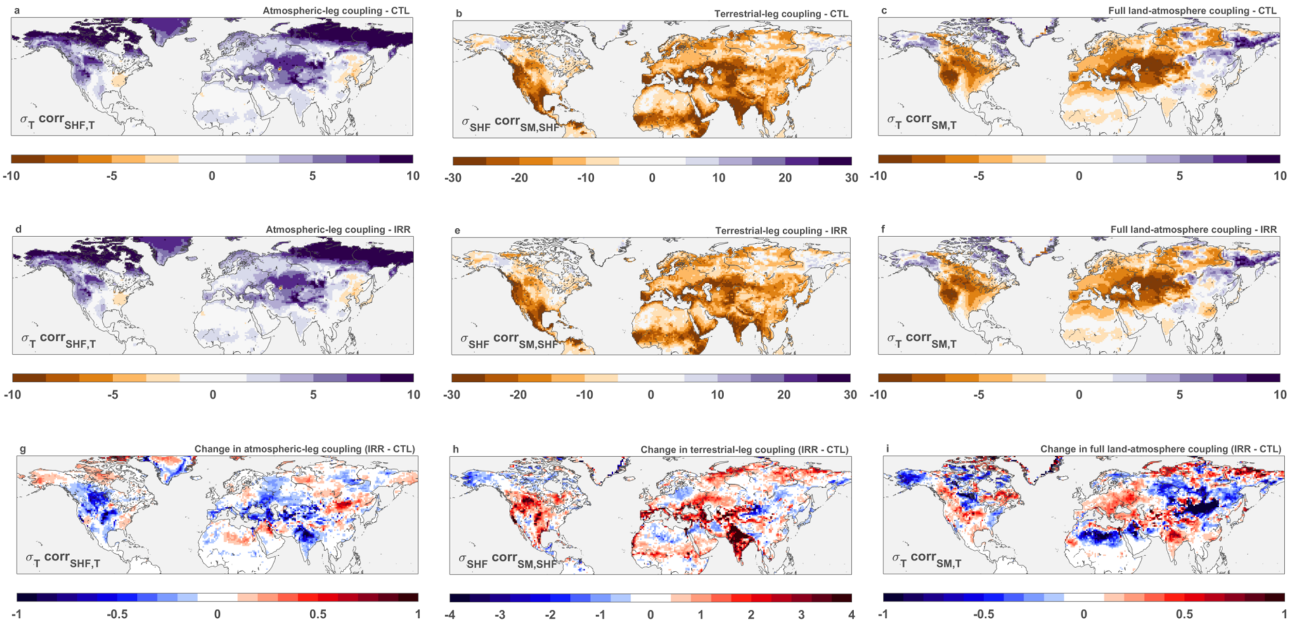

Supplementary Figure 9 | Irrigation influence on land-atmosphere coupling strength reveals reduced temperature dependence on sensible heating and soil moisture. Results from applying the two-legged coupling framework[4] to the CESM simulations. The atmospheric leg, terrestrial leg and full land-atmosphere coupling are shown for **(a-c)** the CTL ensemble, **(d-f)** the IRR ensemble, and **(g-i)** the difference between both ensembles. σ denotes the standard deviation, corr the Pearson correlation coefficient, T the 2-meter air temperature, SHF the sensible heat flux, and SM the soil liquid water and ice in the top 10 cm of the soil. Note the difference in color scale in the second column compared to the first and last column.

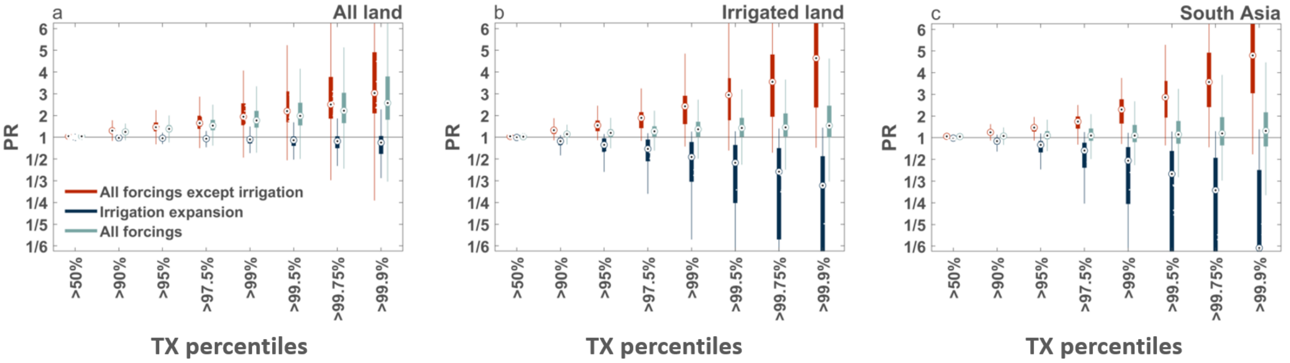

Supplementary Figure 10 | Spatial spread in the masking of trends in hot extremes due to irrigation. Same as Fig. 3, but now showing the boxplots instead of only the median probability ratio (PR) for all land (**a**), all irrigated land (**b**), and South Asia (**c**). Boxplots indicate the spatial PR distribution (center line: median; box limits: upper and lower quartiles) with whiskers extending to the last value located within a distance of 1.5 times the interquartile range from the 25th and 75th quantile, respectively. Outliers are not shown.

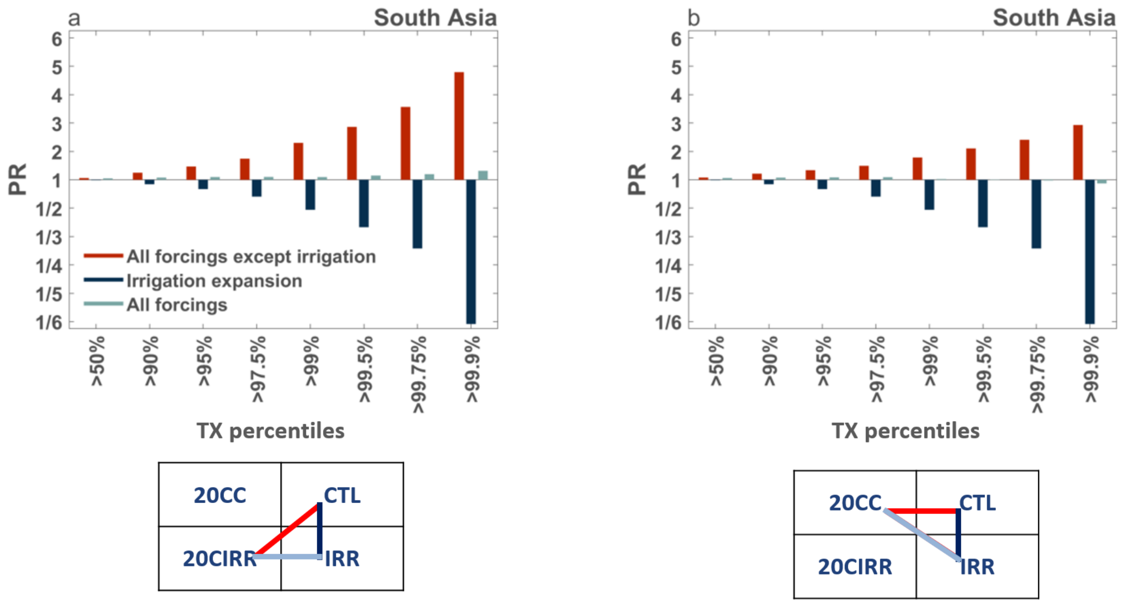

Supplementary Figure 11 | Excluding irrigation in the early 20th century reference run reinforces the conclusions of this study. (**a**) Same as Fig. 3c, i.e. using an early twentieth century model ensemble including irrigation as reference, (**b**) same as Fig. 3c, but now using an early twentieth century model ensemble without irrigation as reference.

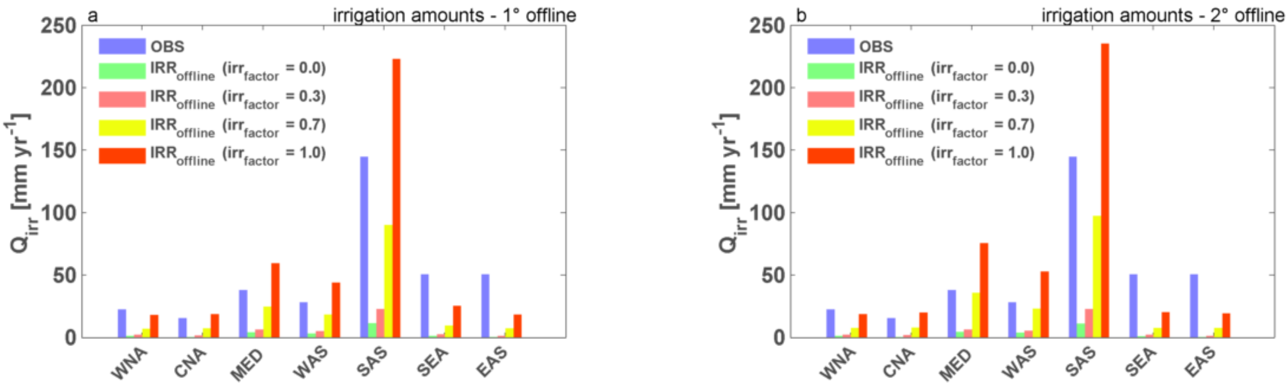

Supplementary Figure 12 | Irrigation amounts for different values of the irrigation factor (see eq. 20.7 in[5]), for (**a**) ∼1° and (**b**) ∼2° land-only simulations with CLM4. Values of the irrigation factor were set to 0 (target soil moisture content = removal of water stress), 0.3, 0.7 (default) and 1.0 (target soil moisture content = saturation soil moisture), respectively.

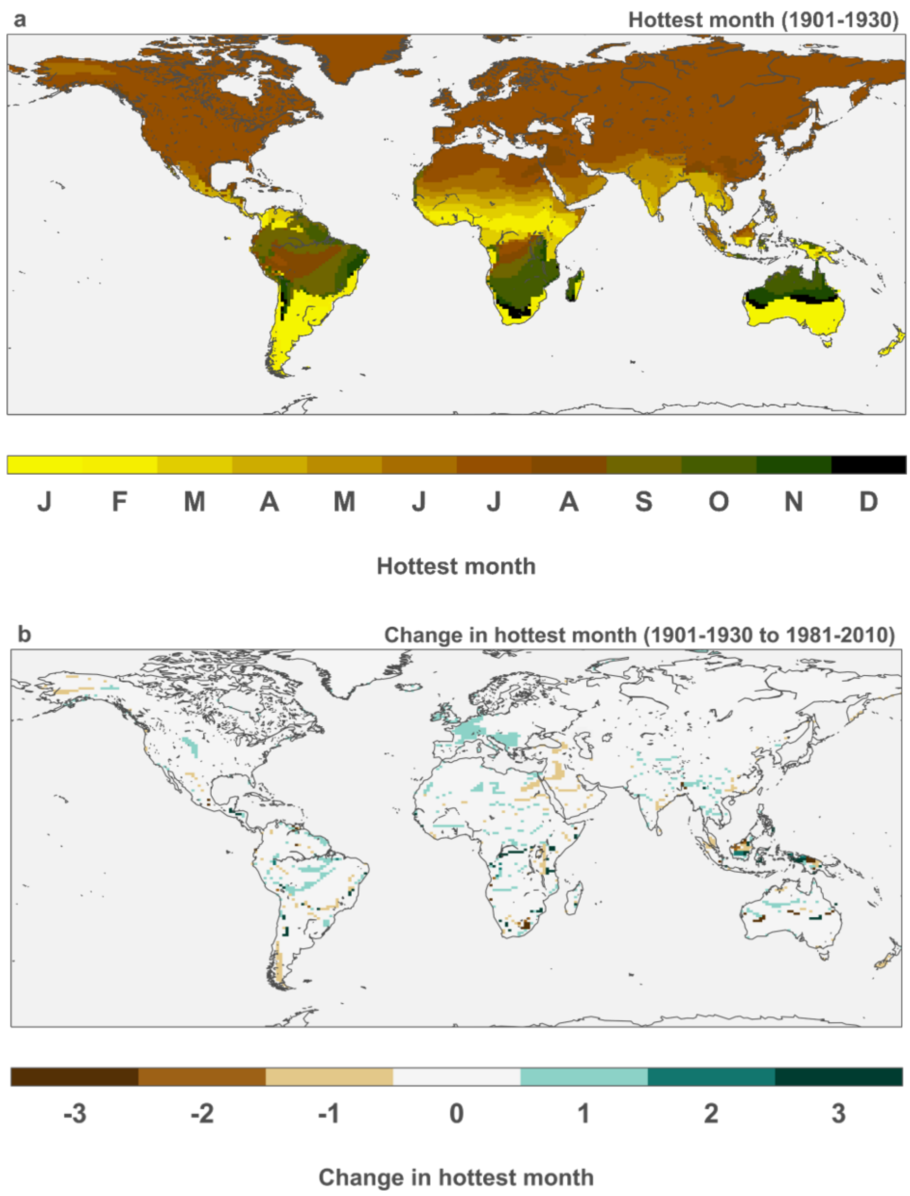

Supplementary Figure 13 | Climate change and irrigation expansion have limited influence on the timing of the hottest month of the year. (**a**), hottest month of the year during the reference period according to the IRR_20C ensemble. (**b**), change in hottest month of the year from the reference period (IRR_20C ensemble) to the present-day period (IRR ensemble).

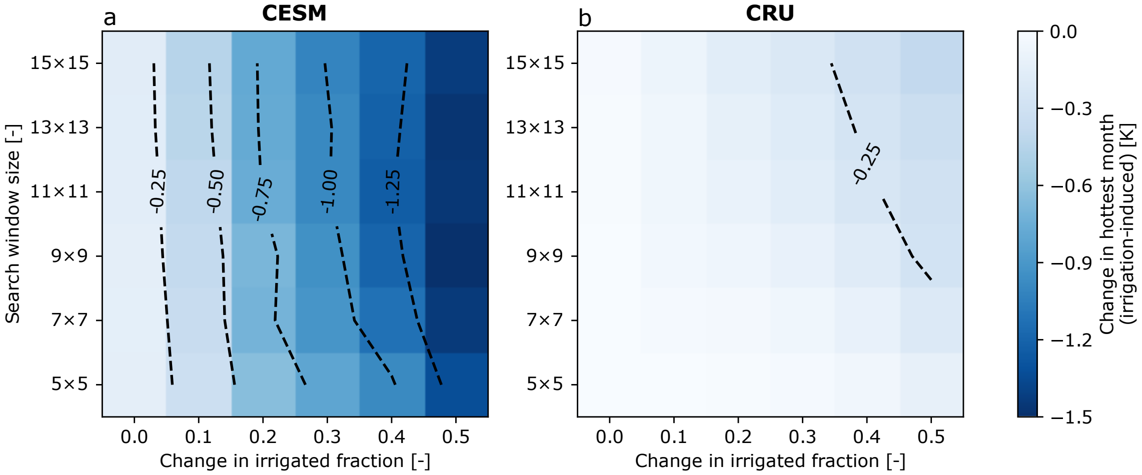

Supplementary Figure 14 | Sensitivity to changes in irrigated fraction dominates over sensitivity to search window size. Irrigation-induced daytime temperature change during the hottest month of the year across a range of sensitivity experiments with window size varying from 5×5 to 15×15 (y-axis). Results are binned according to the change in irrigated fraction (x-axis) and bin medians are shown for (**a**) CESM and (**b**) CRU.

Supplementary Notes

Supplementary Note 1: Challenges for observational analyses

Although gridded data sets dedicated to temperature extremes exists[6,7], we did not consider these data sets here as they have much coarser resolution and often lack data in irrigation hotspots[8,9]. But also analyses based on the CRU data set need to be interpreted with caution, since the data set has a global coverage by assuming a spatial footprint of climate indices (i.e. a range within which information can be drawn from a point observation) and subsequently interpolating station observations to a regular grid[10]. This potentially results in spatial dependence between neighbouring grid cells. In turn, this may possibly explain why, after removing the $\Delta TXm_{\mathrm{irr}}$ signal from ΔTXm, there is still some negative dependence in the observational signal, but not in the model results where the signal is spatially independent (Supplementary Fig. 2). A second point of consideration is the arithmetic nature of the maximum monthly temperature product in CRU. This metric, used to calculated the average maximum temperature during the hottest month of the year (TXm), is obtained as the sum of the mean temperature and half of the diurnal temperature range.

Secondly, we have analysed the sensitivity of the irrigation-induced temperature change to the search window size for CESM and CRU. While the obtained irrigation-induced cooling demonstrates some sensitivity to the search window size, this sensitivity is substantially smaller than the sensitivity to the change in irrigated fraction (Supplementary Fig. 14), confirming the overall validity of our approach. Overall we believe to have selected the right window size considering the coarse resolution of the underlying products and the need to have a large enough window size to derive robust coefficient estimates.

Finally, two considerations should be borne in mind regarding the applicability of the HID data set to quantify temperature effects of irrigation. First, the data set is compiled based on installed irrigation infrastructure rather than actual irrigated land[1]. The possibility of unused irrigation infrastructure in certain areas implies that the method may falsely attribute temperature trends to irrigation. An additional consideration is that spatially aggregating HID data to the CESM resolution may remove part of the spatial signal in irrigated area.

Supplementary Note 2: Comparison against estimates based on remote sensing

Introduction. To test the quality of CESM and the robustness of the window searching algorithm, we applied the algorithm to land surface temperatures (LST) from MODIS and the CESM IRR ensemble. We note that there are some fundamental differences between the CESM comparison to CRU (hereafter referred to as the CRU analysis) and the new analysis on CESM and MODIS (hereafter referred to as the MODIS analysis):

1. When analysing MODIS, we consider only the spatial signal, not the spatiotemporal signal, that is, we look at the difference in present-day absolute LST between irrigated and surrounding non-irrigated regions, rather that at how the local temporal change signal differs between irrigated and surrounding non-irrigated regions (as in the CRU analysis).
2. The MODIS analysis considers LST instead of near-surface air temperatures (T).
3. We perform the MODIS analysis both on the grid-scale and subgrid scale model output, whereas the CRU analysis is performed at the grid-scale only (this is justified by the resolution of CRU being much coarser compared to MODIS). See below for a detailed description of the calculations on the subgrid-scale output.
4. The MODIS grid-scale analysis and CRU analysis are performed at the CESM resolution, while the MODIS subgrid-scale analysis is performed at the spatial resolution of the Historical Irrigation Database (HID).
5. While daily maximum 2m air temperatures can be analysed in CESM, for LST we can only analyse 24h averages from the model due to raw output data limitations.

Methods. The average skin temperatures are calculated for MODIS (based on monthly data from MODIS/Aqua MYD11C3-v006 available at 0.05° spatial resolution[11]) and CESM for 2003-2010, i.e. the years in the MODIS data record with a full annual cycle that overlap with the CESM simulations. In the following, we separate the MODIS analysis into a grid-scale and a subgrid-scale analysis.
Grid-scale analysis: The grid-scale analyses have been performed using a modified version of the window searching algorithm (see Methods section in the main text). We perform the regression on the present-day total LST signal for the months March-May (MAM), the dry pre-monsoon season in South Asia. Since this analysis concerns spatial irrigation impacts, we use the present-day irrigated fraction derived from HID as a regressor (see Methods Equation 1). The model results are calculated from LST output, which was computed from the raw LW_{out} output using the Stefan-Boltzmann law. We use MODIS/Aqua observations from the 01:30 h LT and 13:30 h LT overpass (hereafter referred to as nighttime and daytime overpass, respectively) for the calculation of the observed irrigation impacts on LST. These data were regridded to the CESM-grid using second-order conservative remapping[12] before application in the search window algorithm.
Subgrid-scale analysis: the high resolution of the MODIS observations enables a quantification of the irrigation-induced LST change at a finer resolution than the model output. The sub-grid effect is calculated by taking the difference in skin temperature between pairs of irrigated and non-irrigated crop tiles, to calculate the 'potential irrigation-induced cooling' between a fully irrigated pixel and a non-irrigated pixel. This is then multiplied by the irrigated fraction to obtain an estimate of the actual irrigation-induced cooling rate:

$$\Delta LST_{\mathrm{irr}} = f_{\mathrm{irr}} \times (LST_{\mathrm{crop,irrigated}} - LST_{\mathrm{crop,rainfed}})$$

The sub-grid irrigation effect in MODIS is calculated by application of the search window algorithm to MODIS LST observations (mean of daytime and nighttime overpass when compared to CESM, daytime overpass only when analysed separately), regridded to a 5 arc min resolution of the HID grid. The algorithm configuration is equivalent to that of the grid-scale analysis, but a search window size of 13×13 cells is used to obtain a total search window size of $1.08° \times 1.08°$, so that a search window closely resembles the size of a CESM pixel ($1.25° \times 0.9375°$).
Finally, we note that three caveats imply that there will be an inherent difference between the MODIS- and CESM-based results:

1. Both LST and cloud cover are stored only at monthly time step for our CESM runs. Hence we cannot exclude cloudy days from the CESM output. As such, the MODIS data contains only clear-sky values whereas the CESM output contains all-sky values.

2. We assume that emissivity equals 1 when converting the raw CESM LW_{out} values to LST using the Stefan-Boltzmann law. In reality, emissivity may differ from 1. Moreover, the resulting LST values are sensitive to the choice of the emissivity value.

3. For MODIS, we take the monthly mean of the average of the daytime and nighttime overpasses, which correspond more or less to the daily maximum and daily minimum, respectively, whereas for CESM we can only work with the monthly mean of the 24h average as the raw LW_{out} data is only stored at this temporal resolution.

Results. The absolute LST values as simulated by CESM correspond reasonably well to MODIS values (Supplementary Fig. 4a). A cold bias during the winter months is consistent with two inherent differences between CESM and MODIS listed above: no cloud masking and an emissivity of 1 both tend towards a cold bias in CESM. Given the current model set-up it is however difficult to determine which of these effects is the dominant factor in explaining biases in CESM.
Application of the spatial window-searching method to CESM and MODIS LST (grid-scale) indicates that CESM underestimates the irrigation-induced cooling relative to MODIS (Supplementary Fig. 4b). We note that this is opposite to the response obtained from applying the spatio-temporal window-searching method to CESM CRU T (grid-scale), where CESM overestimates the irrigation signal compared to CRU (Figure 1). Overall this suggests the presence of substantial uncertainties in both model and observational products, but also highlights differences in the response of LST versus T to irrigation. The latter is consistent with recent results for another land surface forcing[13] showing that both in models and observations, near-surface air temperatures respond very differently to deforestation compared to land surface temperatures. Despite the uncertainty in the magnitude of the cooling, the signal of the irrigation-induced effect on LST is remarkably consistent between CESM and MODIS in terms of sign (Supplementary Fig. 4c): only 18 out of 326 considered pixels show an opposite sign in their response to irrigation (5.52%). Supplementary Fig. 4c furthermore shows that both in CESM and MODIS the strongest cooling occurs for pixels with the highest present-day irrigated fraction. From

these results we conclude that CESM may be used a tool to investigate irrigation effects on surface climate, but that absolute values need to be interpreted with caution.

The irrigation-induced cooling during daytime as retrieved from MODIS for South Asia has a marked seasonality, with the strongest cooling during the months March-May (MAM) and hardly any effect during June-August (JJA; Supplementary Fig. 5b). This is consistent with earlier results[8] showing the strongest cooling effect for LST in CESM during MAM and a rapid removal of the signal around the onset of the Indian Monsoon. This reinforces our earlier conclusion that CESM may be used in this context.

Finally, comparison of the MODIS window-searching method to the CESM subgrid-scale effect shows a relatively consistent median response in model and observations over South Asia but a larger spatial variability in MODIS (Supplementary Fig. 6). Probably this difference in variability is owing to the large difference in spatial resolution between CESM and MODIS.

Discussion. The above analysis highlights the challenges of comparing models and observation characterised by uncertainties, but at the same time demonstrates that CESM may be used as a tool for robust irrigation impact assessments. Furthermore, we believe that it is difficult to compare irrigation-induced changes in T and LST. This is consistent with recent findings[13] that LST and T may respond differently to deforestation in Earth system models. Consequently, deriving irrigation-induced changes in T based on LST is not straightforward, and we have therefore not attempted such a conversion here. Future research may however further investigate the potential of remote sensing to derive changes in near-surface air temperature from LST data[14], and explore the potential of using subgrid-scale output from multiple Earth system models to derive robust biogeophysical responses to land cover changes.

Supplementary Note 3: Irrigation influence on land-atmosphere coupling strength

To quantify the simulated effect of irrigation on land-atmosphere coupling strength, we apply the two-legged coupling framework[4,15] to our CESM simulations. In particular, we use daily model output for three variables: 2-meter air temperature (T), sensible heat flux (SHF) and soil moisture (liquid+solid) in the top 10 cm of the soil (SM). Using these variables we compute three metrics: (i) the atmospheric leg of the coupling, defined here as the product of the standard deviation of T and the correlation between T and SHF (σ_T corr$_{SHF,T}$), (ii) the terrestrial leg of the coupling, defined here as the product of the standard deviation of SHF and the correlation between SHF and SM (σ_{SHF} corr$_{SM,SHF}$), and (iii) full land-atmosphere coupling, defined here as the product of the standard deviation of T and the correlation between SM and T (σ_T corr$_{SM,T}$). All metrics were computed on the native CESM grid, at daily resolution for each individual year, and subsequently averaged across all 30 analysis years and ensemble members. Since most irrigation areas are located in the northern hemisphere, we focus our analysis on northern hemisphere and the months March-August (MAMJJA; Supplementary Fig. 9), thus including the hottest months in most of the study area (Supplementary Fig. 13a). While there

are some differences between the two-legged coupling metric and the standard Global Land-Atmosphere Coupling Experiment (GLACE) metric Ω_T[16,17], we note that generally coupling hotspots are similar between the various diagnostics[18].

The sign of each metric denotes regions where the two considered variables are correlated or anti-correlated. Hence the sign reversal between the atmospheric and terrestrial leg of the coupling highlights the general relation between both variables (Atmospheric leg: higher SHF inducing higher T; Terrestrial leg: higher SM inducing lower SHF), whereas the magnitude is indicative of the strength of the coupling, with values closer to zero indicating weaker coupling. However, overall one should be careful with the interpretation of the results of this analysis at the local to regional scale, as well as with the attribution of irrigation effects outside the main irrigation centers, considering the importance of natural variability in this context.

Over South Asia, both the atmospheric and terrestrial leg of the coupling are weakening due to irrigation activity (that is, in both cases the coupling in IRR is closer to zero compared to CTL, Supplementary Fig. 9a-b,c-d), leading to an overall decrease in full land-atmosphere coupling (Supplementary Fig. 9c,f). This is likely because irrigation leads to a decrease in the variability of SHF. Regarding the atmospheric leg, the decrease is particularly pronounced over South Asia, suggesting that irrigation additionally dampens temperature variability, consistent with earlier findings[8]. Furthermore, timing of irrigation during periods of vegetation water scarcity reduces temporal SM variability and associated terrestrial leg as well as full land-atmosphere coupling.

Finally, irrigation effects on climate as simulated by CESM may be erroneous in case the impact of dry soils – without irrigation – on daily temperature extremes would be overestimated in the model. In this context, we note that CESM does not highlight South Asia as a land-atmosphere coupling hotspot (Supplementary Fig. 9c), in contrast to several other models participating in the GLACE studies[17]. Therefore, it is unlikely that CESM overestimates the impact of dry soils on climate compared to other Earth system models.

Supplementary Note 4: Sensitivity of irrigation amounts to parameter settings

To quantify the possible effect of parameter settings on modelled irrigation quantities, we tested CLM's sensitivity to the only free parameter in the irrigation module, the weighting factor in the target soil moisture calculation (see eq. 20.7 in[5]). In particular, we set it to values 0 (target soil moisture content = removal of water stress), 0.3, 0.7 (default) and 1.0 (target soil moisture content = saturation soil moisture). We ran 33-year offline CLM simulations (1972-2004) using bias-corrected reanalysis data as atmospheric forcing[19] and exclude the first three years from the analysis.

We find high sensitivity of regional irrigation quantities to the irrigation factor in most regions (Supplementary Fig. 12). In South Asia (SAS), a change in the irrigation factor leads to strong biases in modelled irrigation quantities (and assumed associated biases in climatic impacts

in case the simulations would have been run in coupled mode). We note however that this parameter was originally set to match global observed irrigation quantities around the year 2000, which is confirmed by evaluation of our IRR simulation[8]. We also note that this sensitivity is independent of model resolution: both the $\sim$1° and $\sim$2° offline simulations demonstrate similar sensitivities (Supplementary Fig. 12). We therefore conclude that irrigation quantities in most regions, and notably in the rice paddy fields of South Asia, are adequately reproduced by keeping the only free parameter in the irrigation module to its default value.

Supplementary References

[1] Siebert, S. *et al.* A global data set of the extent of irrigated land from 1900 to 2005. *Hydrology and Earth System Sciences* **19**, 1521–1545 (2015).

[2] Doxsey-Whitfield, E. *et al.* Taking Advantage of the Improved Availability of Census Data: A First Look at the Gridded Population of the World, Version 4. *Papers in Applied Geography* **1**, 226–234 (2015).

[3] Seneviratne, S. I. *et al.* Changes in climate extremes and their impacts on the natural physical environment. In Field, C. *et al.* (eds.) *Managing the Risks of Extreme Events and Disasters to Advance Climate Change Adaptation*, 109–230 (Cambridge University Press, Cambridge, UK, 2012).

[4] Dirmeyer, P. A., Wang, Z., Mbuh, M. J. & Norton, H. E. Intensified land surface control on boundary layer growth in a changing climate. *Geophysical Research Letters* **41**, 1290–1294 (2014).

[5] Oleson, K. W. *et al.* Technical description of version 4.5 of the Community Land Model (CLM). *NCAR Technical Note* **1**, 420 (2013).

[6] Donat, M. G. *et al.* Global land-based datasets for monitoring climatic extremes. *Bulletin of the American Meteorological Society* **94**, 997–1006 (2013).

[7] Donat, M. G. *et al.* Updated analyses of temperature and precipitation extreme indices since the beginning of the twentieth century: The HadEX2 dataset. *Journal of Geophysical Research: Atmospheres* **118**, 2098–2118 (2013).

[8] Thiery, W. *et al.* Present-day irrigation mitigates heat extremes. *Journal of Geophysical Research Atmospheres* **122**, 1403–1422 (2017).

[9] Hirsch, A. L. *et al.* Modelled biophysical impacts of conservation agriculture on local climates. *Global Change Biology* **24**, 4758–4774 (2018).

[10] Harris, I., Jones, P. D., Osborn, T. J. & Lister, D. H. Updated high-resolution grids of monthly climatic observations - the CRU TS3.10 Dataset. *International Journal of Climatology* **34**, 623–642 (2014).

[11] Wan, Z. New refinements and validation of the MODIS Land-Surface Temperature/Emissivity products. *Remote Sensing of Environment* **112**, 59–74 (2008).

[12] Jones, P. W. First- and Second-Order Conservative Remapping Schemes for Grids in Spherical Coordinates. *Monthly Weather Review* **127**, 2204–2210 (1999).

[13] Winckler, J. *et al.* Different response of surface temperature and air temperature to deforestation in climate models. *Earth System Dynamics Discussions* **October**, 1–17 (2019).

[14] Hooker, J., Duveiller, G. & Cescatti, A. Data descriptor: A global dataset of air temperature derived from satellite remote sensing and weather stations. *Scientific Data* **5**, 1–11 (2018).

[15] Dirmeyer, P. A. The terrestrial segment of soil moisture-climate coupling. *Geophysical Research Letters* **38**, 1–5 (2011).

[16] Seneviratne, S. I. *et al.* Impact of soil moisture-climate feedbacks on CMIP5 projections: First results from the GLACE-CMIP5 experiment. *Geophysical Research Letters* **40**, 5212–5217 (2013).

[17] Guo, Z. *et al.* GLACE: The Global Land-Atmosphere Coupling Experiment. Part II: Analysis. *Journal of Hydrometeorology* 611–625 (2006).

[18] Lorenz, R. *et al.* Influence of land-atmosphere feedbacks on temperature and precipitation extremes in the GLACE-CMIP5 ensemble 607–623 (2015).

[19] Qian, T., Dai, A., Trenberth, K. E. & Oleson, K. W. Simulation of Global Land Surface Conditions from 1948 to 2004. Part I: Forcing Data and Evaluations. *Journal of Hydrometeorology* **7**, 953–975 (2006).
